# Effects of a 12-Week Recreational Skiing Program on Cardio-Pulmonary Fitness in the Elderly: Results from the Salzburg Skiing in the Elderly Study (SASES)

**DOI:** 10.3390/ijerph182111378

**Published:** 2021-10-29

**Authors:** David Niederseer, Roman Walser, Christian Schmied, Flemming Dela, Christoph Gräni, Philipp Bohm, Erich Müller, Josef Niebauer

**Affiliations:** 1 Department of Cardiology, University Heart Center, University Hospital Zurich, University of Zurich, 8091 Zurich, Switzerland; roman11walser@gmail.com (R.W.); Christian.Schmied@usz.ch (C.S.); Philipp.Bohm@gmx.de (P.B.); 2 Institute of Sports Medicine, Prevention and Rehabilitation, Paracelsus Medical University Salzburg, 5020 Salzburg, Austria; j.niebauer@salk.at; 3 Department of Geriatrics, Bispebjerg-Frederiksberg University Hospital, DK-2400NV Copenhagen, Denmark; fdela@sund.ku.dk; 4 Xlab, Department of Biomedicine, University of Copenhagen, DK-2200N Copenhagen, Denmark; 5 Department of Cardiology, Bern University Hospital, 3010 Bern, Switzerland; christoph.graeni@insel.ch; 6 Department of Sport Science and Kinesiology, University of Salzburg, 5020 Salzburg, Austria; erich.mueller@sbg.ac.at

**Keywords:** cardio-pulmonary exercise testing, spirometry, ski, high altitude, elderly, training program

## Abstract

Objectives: To investigate whether recreational alpine skiing in the elderly can improve cardio-pulmonary fitness. Design: Randomized controlled study with pre–post repeated measurements. Methods: A total of 48 elderly participants (60–76 years) were randomly assigned to either participate in a 12-week guided recreational skiing program (intervention group, IG, average of 28.5 ± 2.6 skiing days) or to continue a sedentary ski-free lifestyle (control group, CG). Cardio-pulmonary exercise testing (CPET) and pulmonary function testing were performed in both groups before (PRE) and after (POST) the intervention/control period to compare parameters PRE vs. POST CPET. Results: At baseline, IG and CG did not differ significantly with respect to CPET and pulmonary function parameters. At POST, several measures of maximal exercise capacity and breathing economy were significantly improved in IG as compared to CG: maximal oxygen capacity (IG: 33.8 ± 7.9; CG: 28.7 ± 5.9 mL/min/kg; *p* = 0.030), maximal carbon dioxide production (IG: 36.2 ± 7.7; CG: 31.8 ± 6.5 mL/min/kg; *p* = 0.05), maximal oxygen pulse (IG: 16.8 ± 4.2; CG: 13.2 ± 4 mL/heart beat; *p* = 0.010), maximal minute ventilation (IG: 96.8 ± 17.8; CG: 81.3 ± 21.9 l/min; *p* = 0.025), and maximal metabolic equivalent of task (METs, IG: 9.65 ± 2.26; CG: 8.19 ± 1.68 METs; *p* = 0.029). Except for oxygen pulse, these significant changes could also be observed at the anaerobic threshold. Maximal heart rate and pulmonary function parameters remained essentially unchanged. Conclusion: Regular recreational skiing improves cardio-pulmonary fitness along with breathing economy and thus can contribute to a heart-healthy lifestyle for the elderly.

## 1. Introduction

Cardio-pulmonary exercise testing (CPET) is used in medicine in general and in sports medicine as well as sports and exercise science in particular to assess and monitor cardio-pulmonary fitness and training in healthy subjects as well as in patients [1,2].

Maximal oxygen capacity (VO_2max_) is a frequently measured variable in clinical practice to quantify cardio-pulmonary exercise capacity and is often used as the primary outcome parameter in CPET and/or exercise training studies [3,4]. We had previously reported on VO_2max_ as a single parameter used in the assessment of the cardiovascular risk/benefit profile of the participants in the SASES-study [5]; however, a detailed analysis of all CPET findings has not been provided.

It has been shown that healthy elderly participants aged >60 years can improve their physical fitness during a structured program of endurance exercise training, e.g., on treadmills, matching the extent of improvement of younger individuals [6,7]. However, adherence to unmonitored exercise training prescription remains a major problem. Müller et al. were the first to investigate guided alpine skiing for elderly people, as it is a form of exercise, which is conducted by many during the winter season in alpine regions [8].

Alpine skiing requires a moderate to high level of physical fitness and demands both aerobic and anaerobic energy supply. Typically, the bouts of physical activity only last a few minutes with longer breaks in between due to uphill transport. This results in a natural interval training regimen with varying episodes of physical activity and intensity levels [9].

Spirometry is the primary diagnostic test to assess pulmonary function and the primary tool to measure forced vital capacity (FVC) and forced expiratory volume per second (FEV1) with subsequent calculation of the Tiffeneau index (ratio of FEV1 to FVC) and peak expiratory flow (PEF). A meta-analysis in young participants and athletes undergoing specific respiratory muscle training showed a significant effect on physical performance independent from pulmonary function parameters, where no changes were found, indicating a predominance of adaptative mechanisms in the gas exchange system [10]. Deterioration of FEV1, FVC, and PEF with age is known [11], as are pre–post exercise training studies in healthy participants of all ages showing essentially no effect on pulmonary function parameters [12,13]. To the best of our knowledge, the effects of alpine skiing on an elderly person’s pulmonary function has not yet been studied.

The aim of this study was to collect data from CPET and pulmonary function testing before and after a guided alpine skiing program in order to assess whether, and if so, through which mechanisms, cardio-pulmonary changes could be achieved at rest and during maximal exercise.

## 2. Methods

The design of this study has been previously reported elsewhere [14]. Briefly, the study population was randomized into an intervention group (IG, *n* = 29; age: 66.6 ± 2.1 years, 45% female) or a control group (CG, *n* = 19; age: 67.3 ± 4.4 years, 41% female). The IG performed guided skiing for 12 weeks—a common time frame for a winter season in the alpine region—averaging 28.5 ± 2.6 total days and 3.5 h of skiing per day. A detailed analysis of the composition of an average skiing day in rest, lifting time, and actual skiing is provided elsewhere [14]. The CG was asked to maintain their sedentary lifestyle and to refrain from skiing during the study period. A detailed report on the inclusion and exclusion criteria as well as concomitant medication and medical conditions have been previously reported [5,14]. The study protocol conforms to the ethical guidelines of the 1975 Declaration of Helsinki as reflected in a priori approval by the institution’s human research committee (The Ethics Committee of the University of Salzburg), and written informed consent was obtained from each patient (study registration: https://clinicaltrials.gov/show/NCT01248910).

Before (PRE) and after (POST) the intervention/control period, we performed CPET and pulmonary function testing in both groups. First, we performed pulmonary function testing in resting condition. Data were collected using the EasyOne Spirometer (ndd Medical Technologies, Zurich, Switzerland). We determined FVC, FEV1, and PEF, and thereby calculated FEV1/FVC. Data collection in pulmonary function testing met the standard criteria for spirometry execution according to scientific standards to detect pathological patterns [15].

Thereafter, we measured common CPET parameters during an incremental ramp-protocol starting at 50 Watt (W), increasing 10 W every minute until individual physical exertion was reached. Averaged results at (a) rest, (b) the estimated anaerobic threshold (AT, when respiratory exchange ratio equaled 1 [16]), and (c) maximal exertion were reported.

CPET variables comprised power in W, heart rate (HR), respiratory rate (RR), total ventilated air (VE), and tidal volume (V_T_). By using gas-exchange monitoring, oxygen consumption and produced carbon dioxide (VO_2_ and VCO_2_) were assessed; thus, oxygen pulse (O_2_ pulse, ratio of VO_2_ by HR that expresses the amount of oxygen ejected from the left ventricle during systole, a surrogate of ventricular stroke volume [17]) as well as oxygen and carbon dioxide equivalents (EqO_2_ and EqCO_2_, defined as the total amount of air being inhaled to absorb 1 L of O_2_ or CO_2_, respectively [17]) were calculated. Furthermore, we determined the metabolic equivalents of task (MET, defined as multiples of (3.5 mL/min/kg of VO_2_, i.e., resting oxygen consumption) [17]) and respiratory exchange ratio (RER, defined as VCO_2_/VO_2_ [17]).

Exertion criteria were defined as previously suggested by Balady et al. [17]: reaching at least 85% of age-related estimated HR_max_ (i.e., reaching a HR of 85% of 220—age), plateau in VO_2max_ despite increasing work load, or RER reaching/exceeding 1.1. A pathological CPET was defined as VO_2_ at AT < 11 mL/min/kg or maximal VE/VCO_2_-slope > 34, being a risk factor for early cardiovascular death, as defined by Gitt et al. [18].

Descriptive statistics are presented as means ± standard deviations. Statistical significance was set at *p* < 0.05. The Shapiro–Wilk test was used to check for normal distribution in small sample groups. If data were normally distributed, intra-group comparisons were made using paired *t*-tests, whereas inter-group comparisons were performed by unpaired *t*-tests. If data were not normally distributed, we employed the Mann–Whitney U or Wilcoxon signed-rank tests. We used GraphPad InStat (Version 3.06 for Windows, San Diego, CA, USA) and SPSS (2010, SPSS 18.0.2 for Windows, SPSS Inc., Chicago, IL, USA).

## 3. Results

In IG, two out of the 29 participants did not undergo CPET testing due to claustrophobia while wearing the mask. A total of eight participants (IG: 5 CG: 3) had to be excluded from the study because maximal exertion criteria were not reached, resulting in a total number of 22 participants with complete measurements in IG and 17 in CG. Baseline characteristics of the study population (*n* = 39; IG = 22, CG = 17) are reported in Table 1. At baseline, no significant difference in CPET or pulmonary function data was found between groups except for a lower respiratory rate on maximal exertion (IG: 43.6 ± 7.6 vs. CG: 37.4 ± 5.2/min; *p* = 0.008). All participants had a normal age-related cardio-pulmonary fitness and pulmonary function. No pathological patterns in CPET were found. 

**Figure 1:**
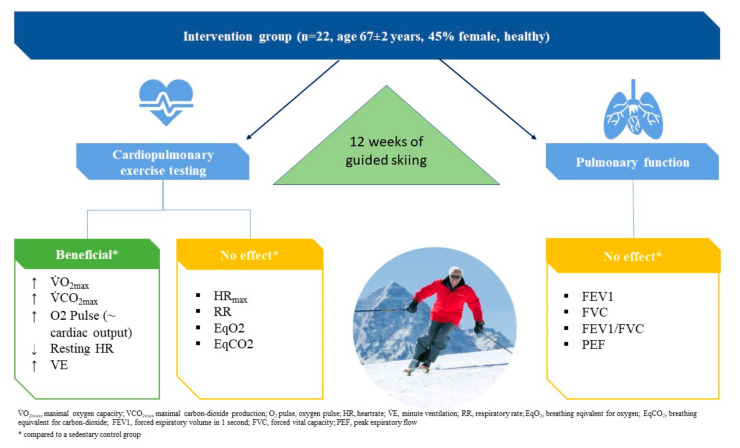
Graphical abstract summarizing the findings of this study.

An overview of CPET measurements is provided in Table 2 and Figure 1. Inside IG, we report significant changes from PRE to POST in VO_2_, VCO_2_, VE, MET, and V_T_, and no changes in HR, O_2_ pulse, RR, W, EqO_2_, and EqCO_2_. CG did not show any changes. IG and CG differed significantly at POST in VO_2_, VCO_2_, O_2_ pulse, MET, RR, and VE. In addition, there was a trend to a lower resting HR in the IG compared to CG (IG vs. CG: Δ 8.3%, *p* = 0.085). Maximal heart rate did not change (IG vs. CG: Δ 3.5%, *p* = 0.643); also, heart rate at AT was not significantly different between the two groups (IG vs. CG: Δ 2.3%, *p* = 0.610).

At POST, absolute VO_2max_ was found to be significantly higher in the IG (2588 ± 651 mL/min) as compared to CG (2093 ± 585 mL/min) corresponding to a difference of 19.0% (*p* = 0.021). In addition, relative VO_2max_ adjusted to body weight differed significantly at POST (IG: 33.8 ± 7.9, CG: 28.7 ± 5.9 mL/min/kg, Δ 15.0%; *p* = 0.015) and at AT (IG: 21.6 ± 3.8, CG: 18.6 ± 3.7mL/min/kg, Δ 13.8%; *p* = 0.013). Consequently, MET also increased (IG: 9.7 ± 2.3, CG: 8.2 ± 1.7, Δ 15.2%; *p* = 0.029). Absolute VCO_2max_ at POST was higher in IG as compared to CG (2787 ± 645 vs. 2314 ± 609 mL/min; Δ 17.0%; *p* = 0.033), also after adjustment for body weight (36.2 ± 7.7 vs. 31.8 ± 6.5 mL/min/kg, Δ 12.0%; *p* = 0.012). This effect was also seen at AT (VCO_2_, 21.6 ± 3.8 vs. 18.6 ± 3.7 mL/min/kg; Δ 13.4%; *p* = 0.010).

At POST, we report no difference between groups in O_2_ pulse at rest (IG vs. CG: *p* = 0.341). However, IG participants showed significantly higher O_2_ pulses at maximal exertion (16.8 ± 4.2 mL/beat in IG, as compared to 13.2 ± 4 mL/beat in CG, *p* = 0.010, Δ 21.5%) and AT (*p* = 0.007, Δ 20.0%).

We observed a significant difference in VE at maximal exertion at POST (IG: 96.8 ± 17.83, CG: 81.3 ± 21.9 l/min; *p* = 0.025). This increase was mainly driven by a more economic ventilation with a significant increase in maximal V_T_ in IG (PRE: 2.1 ± 0.6, POST: 2.3 ± 0.6 l, +10.8%; *p* = 0.032; no changes in CG and no inter-group difference), whereas RR did not change in IG (*p* = 0.805). No changes of VE, V_T_, and RR at resting state were observed.

EqO_2_ did not change at all (*p* = 0.490 at maximal exertion). For EqCO_2_, no changes could be observed at maximal exertion (*p* = 0.616) or AT (*p* = 0.631) but was found higher by trend at resting (33.3 ± 8.1 vs. 29.8 ± 4.2 l/l, Δ 10.5%; *p* = 0.055).

Essentially no changes were observed in pulmonary function measurements throughout the study, as shown in Table 3. Smoking status did not significantly influence our findings (data not shown).

## 4. Discussions

In this study, we demonstrate that guided alpine skiing two to three times per week over the course of 12 weeks in otherwise sedentary elderly participants induced significant changes in the cardio-pulmonary system as assessed by CPET but not in pulmonary function. Most importantly, changes in aerobic capacity, oxygen pulse, and maximal minute ventilation resulting in a better performance during exercise were observed.

Similar results from more traditional exercise training studies with elderly participants have been previously observed; most of them reported VO_2max_ to be the most predictive parameter for exercise capacity [7,19]. Accordingly, an increase in VO_2max_, as we report here, is a well-established finding in exercise training studies. We herewith conclude that alpine skiing can induce a clinical and statistically significant increase in exercise capacity.

Both relative and absolute VO_2max_ increased in IG from PRE to POST (+12%), and there was also a significant difference compared to CG (Δ 15%). Seals et al. and Hagberg et al. [20,21] described similar changes of VO_2max_ after a comparable amount of exercise training (low intensity vs. high intensity at 50–70% and 75–85% of VO_2max_, respectively). Badenhop et al. showed that there were no significant differences between low- and high-intensity training groups after 9 weeks of treadmill training of >60-year-old participants [6]. Another study investigated >60-year-old participants for one year: 6 months with low-intensity unsupervised activity such a walking (40% of VO_2max_, 20–30 min of moderate-intensity walking at least three times per week) and 6 months with high-intensity supervised training (progressively increased exercise training aiming for 45 min at 85% of heart rate reserve during the final 8 weeks of training). This intervention group was compared to a sedentary control group. Seals et al. showed that low-intensity unsupervised training resulted in a significant improvement of VO_2max_; however, supervised high-intensity training resulted in an additional and even greater increase in VO_2max_ [20]. When comparing our reported effect of supervised alpine skiing on VO_2max_ with previous literature on low-, moderate-, and high-intensity exercise training on elderly participants, alpine skiing induced changes best compared to moderate to high-intensity rather than low-intensity exercise training.

VO_2max_ shows a natural decline while aging [19]. Hagberg et al. reported that an age-related loss of aerobic capacity could be delayed by moderate-intensity training, indicating that this also applies to elderly people who ski more frequently. Makrides [4] showed that elderly sedentary participants (60–70 years) were similarly, if not more, capable of raising their relative aerobic power through a twelve-week high-intensity program compared to young sedentary participants (20–30 years), observing not only an increase in VO_2max_ (+38% in elderly vs. 29% in young) but also a maximal estimated cardiac output (+30% vs. +14%) and stroke volume (+21% vs. +9%) compared to baseline. Since a higher VO_2max_ along with a raised O_2_ pulse (a surrogate of the maximal stroke volume and cardiac output in trained and untrained individuals [22]) in elderly participants is related to a significant reduction in cardiovascular and all-cause mortality [23], we conclude hereby a clinically relevant change in the general health prognosis of our study population.

As a consequence of the alpine skiing training regimen, VO_2_ in IG was not only significantly higher at maximal exertion but also at AT, which implies relevant metabolic adaptation already at an earlier stage of exertion. This is in keeping with our previous findings of muscle biopsies in IG, where significant alterations in both structure and metabolism of the musculature [24,25] were seen that are likely responsible for the improvement of both VO_2_ and VCO_2_ at maximal exertion and AT. VCO_2_ at AT and at maximal exertion were higher in IG compared to CG, respectively. We interpret this finding as an adaption toward a more efficient metabolism, indicating a similar capability of the body to dispose acidotic equivalents.

The increased oxygen demand in the musculature during exercise is predominantly supplied by an increase in cardiac output. As HR_max_ is limited by age, gender, and genetics with only low interference of fitness effects [26], neither relevant changes in heart rate at exercise between or within the groups nor at AT or on maximal exertion were observed. Since HR did not change, the enhanced cardiac output is a result not only of an increased stroke volume but also of a VO_2_-mediated increase in oxygen content inside every ejected stroke, surrogated by the O_2_ pulse, which was 22% higher in IG than CG.

Increased VE_max_ and at AT is connected to a significantly greater V_T_, as seen in IG from PRE to POST. Given the similar baseline parameters in IG and CG, a larger tidal volume, triggered by a higher exercise demand, suggests a better ventilatory efficiency and has greater effect on gas exchange than an increased respiratory rate [27]. It is a core and easy-to-monitor mechanism to maintain the VO_2_-driven cellular oxygen supply.

Furthermore, especially, the identification of the lowest EqCO_2_ (VE/VCO_2_) has been observed to be a strong predictor for cardiovascular death [28]. We report changes in VCO_2_, VO_2_, and VE, but since these changes were positively correlating with each other, no changes in EqCO_2_ or EqO_2_ could be observed.

Good fitness levels correspond to a general health benefit preventing cardiovascular [5] and many other diseases elderly people are prone to get and result in an overall improved quality of life, well-being, and longevity [29]. By improving modifiable cardiovascular risk factors, a comparable outcome as compared to common cardiac rehabilitation programs can be assumed [30,31].

Data on pre–post exercise training on pulmonary function parameters are scarce [12,13]. In accordance with the available literature, we report no essential inter-group differences in pulmonary function parameters. To further stress this point, we here report significant and clinically relevant changes in CPET parameters but not in pulmonary function, both of which are in keeping with previously reported findings in traditional exercise training studies in elderly subjects. Unlike many other studies, we assessed pulmonary function data alongside CPET parameters, which certainly helped with understanding the single influencing mechanisms on individual performance. As to the specific environmental characteristics of alpine skiing (intermittent altitude changes and cold ambient temperature), our findings broaden the current knowledge of pulmonary function and responsiveness to exercise training under these circumstances. Consequently, we can conclude that the reported changes in CPET parameters most likely are the effect of adaptation of the heart and musculature and to a lesser extent of pulmonary function. Unlike other training studies, we measured pulmonary function in our subjects simultaneously to CPET parameters, elucidating the mechanisms on how the elderly increase their exercise capacity.

Intermittent hypoxia is a natural effect of alpine skiing using skiing lifts as the uphill transportation mode. The subjects take, e.g., a cable car to be transported uphill and then ride downhill in a repetitive fashion. As the participants of our study were tracked using GPS sensors, the average altitude covered per skiing day could be calculated. Müller et al. report an average of 4885 ± 816 vertical downhill meters per skiing day that comprised of 9.3 ± 3.0 downhill runs per skiing day [14]. As the maximal altitude of the skiing area where the skiing intervention was performed is 2000 m, an exposure to high altitude (defined as altitude > 2500 m) did not take place. The possible effect of intermittent hypoxia at lower altitudes are likely to be minimal. Rossi et al. discussed this issue extensively in the context of recreational alpine skiing [32].

To the best of our knowledge, this is the first randomized study to comprehensively investigate CPET and pulmonary function data in a pre–post repeated measurement design in recreational skiers of advanced age. Rossi et al. have previously discussed all studies on recreational alpine skiing, including SASES [32]. However, several limitations apply. A total of 10 (IG: 7, CG: 3) participants had to be excluded from the analysis due to lack of fulfilling the exertion criteria (*n* = 8) or due to claustrophobia while wearing the CPET mask (*n* = 2). However, these are aspects that are known as real-world problems in CPET. Finally, our study did not account for the long-term sustainability of our findings. Furthermore, our study is limited due to a relatively small sample size. However, due to the huge effort to facilitate a guided skiing intervention for 12 weeks for 2 to 3 days per week and the significant changes we herewith report, we think that our sample size is reasonable to draw meaningful and clinically relevant conclusions and that a larger sample size would result in similar results. Up until now, to the best of our knowledge, this is the only randomized controlled study on supervised recreational alpine skiing in the elderly; however, we certainly look forward to further research on this topic with more included subjects to further deepen our understanding of the beneficial effects of alpine skiing in the elderly.

## 5. Conclusions

In conclusion, we demonstrate that a season, i.e., 12 weeks, with two to three recreational days of skiing per week performed by elderly skiers has a positive impact on the cardio-pulmonary fitness, in part due to improved breathing economy, and can thereby contribute to a heart-healthy lifestyle.

Practical implications follow:

- In the elderly, regular recreational alpine skiing has similar effects on physical fitness as has been previously reported in moderate-to-high endurance training programs.
- While breathing economy, oxygen pulse, and oxygen consumption during exercise were significantly improved after alpine skiing, pulmonary function remained essentially unchanged.
- Downhill skiing is a potent and attractive exercise modality to improve cardio-pulmonary fitness in the elderly in alpine regions.

## Figures and Tables

**Table 1:** Baseline characteristics of participants and baseline measurements of cardio-pulmonary exercise testing: groups did not differ significantly at baseline.

**Parameter**	**Unit**	**Intervention Group**			**Control Group**					
Age	years	66.8 ± 2.0			67.8 ± 4.3					
Sex	*n*/%	m 12 (55%); f 10 (45%)			m 10 (59%); f 7 (41%)					
BMI	kg/m^3^	27.0 ± 3.4			25.6 ± 2.0					
Current/former/never smokers	*n*/%	2 (9%)/7 (32%)/13 (59%)			0 (0%)/6 (35%)/11 (65%)			***p*-values**		
		**REST**	**AT**	**MAX**	**REST**	**AT**	**MAX**	**REST**	**AT**	**MAX**
HR	bpm	70 ± 12	118 ± 17	155 ± 13	77 ± 13	128 ± 17	168 ± 21	0.066	0.095	0.119
VO_2_	mL/min	383 ± 144	1467 ± 495	2376 ± 854	400 ± 136	1392 ± 459	2174 ± 600	0.19	0.818	0.386
VO_2_*kg^−1^	mL/min/kg	4.9 ± 1.7	18.8 ± 6.3	29.9 ± 8.6	5.4 ± 1.6	18.7 ± 3.7	29.5 ± 6.3	0.114	0.863	0.798
VCO_2_	mL/min	330 ± 140	1470 ± 492	2524 ± 778	372 ± 165	1396 ± 456	2320 ± 579	0.302	0.794	0.291
VCO_2_*kg^−1^	mL/min/kg	4.2 ± 1.6	18.9 ± 6.2	32.0 ± 8.6	5.2 ± 2.6	18.7 ± 3.7	31.6 ± 6.4	0.187	0.889	0.687
O_2_-pulse	mL	5.6 ± 1.9	12.5 ± 4.2	15.5 ± 5.9	5.4 ± 2.1	10.9 ± 3.6	13.0 ± 3.4	0.376	0.218	0.124
MET	3.5 mL/min/kg	1.6 ± 0.9	5.4 ± 1.8	8.5 ± 2.5	1.5 ± 0.5	5.3 ± 1.1	8.4 ± 1.8	0.117	0.865	0.793
RR	x/min	20.4 ± 5.3	24.5 ± 6.0	43.6 ± 7.6	20.3 ± 5.5	21.5 ± 6.7	37.4 ± 5.2	0.572	0.419	0.008 *
VE	l/min	14.1 ± 7.9	46.4 ± 16.8	88.0 ± 24.5	15.5 ± 10.7	43.1 ± 18.4	81.7 ± 22.7	0.463	0.845	0.42
V_T_	l	0.8 ± 0.5	1.9 ± 0.6	2.1 ± 0.6	0.8 ± 0.4	2.1 ± 0.6	2.2 ± 0.6	0.989	0.552	0.505
EqO_2_	l/l	26.6 ± 5.8	29.8 ± 2.7	46.3 ± 5.2	23.8 ± 5.3	28.8 ± 3.2	45.5 ± 11.0	0.139	0.684	0.874
EqCO_2_	l/l	32.7 ± 6.8	29.7 ± 2.7	39.7 ± 4.5	30.3 ± 6.8	28.7 ± 3.2	41.1 ± 10.4	0.402	0.748	0.383

BMI, body mass index; HR, heart rate; VO_2_, oxygen capacity; VO_2_*kg^−1^, oxygen capacity per kilogram; VCO_2_, carbon dioxide production; VCO_2_*kg^−1^, carbon dioxide production per kilogram; O_2_ pulse, oxygen pulse; MET, metabolic equivalent of task; RR, respiratory rate; VE, minute ventilation; V_T_, tidal volume; EqO_2_, breathing equivalent for oxygen; EqCO_2_, breathing equivalent for carbon dioxide; bpm, beats per minute; * statistically significant.

**Table 2:** CPET-results at REST, AT, and MAX in PRE–POST comparison.

Parameter	Unit	Intervention Group (*n* = 22)			Control Group (*n* = 17)			IG vs. CG POST	
		PRE	POST	*p*-Value	PRE	POST	*p*-Value	*p*-Value	Δ Mean
**REST**									
**HR**	bpm	70 ± 12	67 ± 10	0.307	76 ± 12	73 ± 9	0.136	0.085	−8%
**VO_2_**	mL/min	383 ± 144	449 ± 171	0.862	400 ± 135	450 ± 177	0.101	0.497	−0.2%
**VO_2_** *kg^−1^	mL/min/kg	4.9 ± 1.7	5.8 ± 2.3	0.68	5.4 ± 1.6	6.3 ± 2.5	0.102	0.666	−8%
**VCO_2_**	mL/min	330 ± 140	353 ± 128	0.877	371 ± 165	350 ± 140	0.12	0.484	+0.6%
**VCO_2_** *kg^−1^	mL/min/kg	4.2 ± 1.6	4.6 ± 1.7	0.954	5.2 ± 2.6	4.9 ± 1.9	0.122	0.690	−7%
**O_2_ pulse**	mL	5.6 ± 1.9	6.9 ± 2.8	0.476	5.36 ± 2.07	6.3 ± 2.8	0.253	0.341	+8%
**MET**	3.5 mL/kg/min	1.6 ± 0.9	1.6 ± 0.7	0.669	1.5 ± 0.5	1.8 ± 0.7	0.106	0.659	−10%
**RR**	x/min	20 ± 5	20 ± 5	0.147	20 ± 6	17 ± 3	0.683	0.648	+15%
**VE**	l/min	14 ± 8	13.1 ± 4.8	0.593	15.5 ± 10.7	12.7 ± 4.2	0.072	0.328	+3%
**V_T_**	l	0.8 ± 0.5	0.7 ± 0.3	0.788	0.8 ± 0.4	0.8 ± 0.2	0.449	0.67	−10%
**EqO_2_**	l/l	26.6 ± 5.8	26.4 ± 6.6	0.747	23.8 ± 5.3	23.4 ± 3.9	0.247	0.053	+11%
**EqCO_2_**	l/l	32.7 ± 6.8	33.3 ± 8.1	0.916	30.3 ± 6.8	29.8 ± 4.2	0.154	0.055	+11%
**AT**									
**HR**	bpm	118 ± 17	120 ± 16	0.408	128 ± 16	122 ± 16	0.03 *	0.61	−2%
**Watt**	W	105 ± 31	112 ± 30	0.115	95 ± 40	94 ± 32	0.556	0.199	+16%
**VO_2_**	mL/min	1467 ± 495	1655 ± 356	0.036 *	1391 ± 459	1354 ± 353	0.909	0.116	+18%
**VO_2_** *kg^−1^	mL/kg	18.8 ± 6.3	21.6 ± 3.8	0.011 *	18.7 ± 3.7	18.6 ± 3.7	0.579	0.013 *	+14%
**VCO_2_**	mL/min	1469 ± 492	1667 ± 350	0.029 *	1396 ± 455	1360 ± 353	0.832	0.105	+18%
**VCO_2_** *kg^−1^	mL/min/kg	18.9 ± 6.2	21.6 ± 3.8	0.012 *	18.7 ± 3.7	18.7 ± 3.7	0.496	0.01 *	+13%
**O_2_ pulse**	mL	12.5 ± 4.2	13.9 ± 3.1	0.075	10.9 ± 3.6	11.1 ± 3	0.529	0.007 *	+20%
**MET**	3.5 mL/kg/min	5.4 ± 1.8	6.2 ± 1.1	0.011 *	5.3 ± 1.1	5.3 ± 1.1	0.588	0.154	+14%
**RR**	x/min	24 ± 6	24 ± 6	0.911	22 ± 7	23 ± 7	0.157	0.945	+5%
**VE**	l/min	46.4 ± 16.8	52.4 ± 11.1	0.033	43.1 ± 18.4	42.7 ± 13.5	0.635	0.28	+19%
**V_T_**	l	1.9 ± 0.6	2.21 ± 0.48	0.068	2.1 ± 0.6	1.9 ± 0.4	0.068	0.05 *	+14%
**EqO_2_**	l/l	29.8 ± 2.7	30.6 ± 2.6	0.409	28.8 ± 3.2	29.4 ± 3.7	0.147	0.948	+2.5%
**EqCO_2_**	l/l	29.7 ± 2.7	29.9 ± 2.6	0.241	28.7 ± 3.21	29.3 ± 3.19	0.241	0.973	+2.2%
**MAX**									
**HR**	bpm	154 ± 12	155 ± 14	0.921	168 ± 21	160 ± 13	0.092	0.643	−4%
**Watt**	W	173 ± 44	175 ± 47	0.724	152 ± 45	151 ± 45	0.367	0.114	+14%
**VO_2_**	mL/min	2375 ± 854	2588 ± 651	0.09	2173 ± 599	2093 ± 585	0.242	0.021 *	+19%
**VO_2_** *kg^−1^	mL/min/kg	29.9 ± 8.6	34 ± 8	0.015 *	29.5 ± 6.3	28.71 ± 5.9	0.441	0.03 *	+15%
**VCO_2_**	mL/min	2523 ± 777	2787 ± 645	0.033	2320 ± 578	2313 ± 608	0.644	0.023 *	+17%
**VCO_2_** *kg^−1^	mL/min/kg	32.0 ± 8.6	36 ± 8	0.012 *	31.6 ± 6.4	31.8 ± 6.5	0.387	0.051	+12%
**O_2_ pulse**	mL	15.5 ± 5.9	17 ± 4	0.152	13 ± 3.4	13.2 ± 4	0.632	0.01 *	+22%
**MET**	3.5 mL/kg/min	8.5 ± 2.5	10 ± 2	0.015 *	8.4 ± 1.8	8.2 ± 1.7	0.417	0.029 *	+15%
**RR**	x/min	43 ± 7	43 ± 7	0.805	37 ± 5	38 ± 8	0.494	0.037 *	+11%
**VE**	l/min	88.0 ± 24.5	97 ± 18	0.017 *	81.7 ± 22.7	81.3 ± 21.9	0.833	0.025 *	+16%
**V_T_**	l	2.1 ± 0.6	2 ± 1	0.032 *	2.2 ± 0.6	2.2 ± 0.6	0.738	0.441	+6%
**EqO_2_**	l/l	46.3 ± 5.2	47 ± 6	0.406	45.5 ± 11	50.0 ± 6.2	0.390	0.49	−7%
**EqCO_2_**	l/l	39.7 ± 4.5	42 ± 7	0.616	41.1 ± 10.3	42.5 ± 8	0.866	0.527	−2%

* Statistically significant.

**Table 3:** Pulmonary function testing. IG and CG PRE.

Parameter	Unit	Intervention Group			Control Group			IG vs. CG POST
		PRE	POST	*p*-Value	PRE	POST	*p*-Value	*p*-Value
**FEV1**	l	2.91 ± 0.63	2.91 ± 0.53	0.996	2.74 ± 0.63	2.62 ± 0.57	0.039	0.107
**FVC**	l	3.95 ± 0.85	3.77 ± 0.79	0.010 *	3.62 ± 0.77	3.52 ± 0.76	0.037 *	0.318
**FEV1/FVC**	l/l	74.0 ± 5.5	77.7 ± 3.7	<0.001 *	75.5 ± 5.6	74.7 ± 5.6	0.683	0.061
**PEF**	l/s	6.77 ± 1.76	6.83 ± 1.82	0.803	6.04 ± 1.53	6.26 ± 2.16	0.507	0.363

IG, intervention group; CG, control group; PRE, pre-intervention testing; POST, post-intervention testing; FEV1, forced expiratory volume in 1 s; FVC, forced vital capacity; PEF, peak expiratory flow; * statistically significant.

## Data Availability

Data is contained within the article.
